# Emerging role of nanosuspensions in drug delivery systems

**DOI:** 10.1186/s40824-020-0184-8

**Published:** 2020-01-15

**Authors:** Shery Jacob, Anroop B. Nair, Jigar Shah

**Affiliations:** 1 Department of Pharmaceutical Sciences, College of Pharmacy, Gulf Medical University, Ajman, UAE; 2 Department of Pharmaceutical Sciences, College of Clinical Pharmacy, King Faisal University, Al-Ahsa, Saudi Arabia; 3 Department of Pharmaceutics, Institute of Pharmacy, Nirma University, Ahmedabad, Gujarat India

**Keywords:** Nanosuspension, Manufacturing methods, Formulation components, Drug delivery systems

## Abstract

Rapid advancement in drug discovery process is leading to a number of potential new drug candidates having excellent drug efficacy but limited aqueous solubility. By virtue of the submicron particle size and distinct physicochemical properties, nanosuspension has the potential ability to tackle many formulation and drug delivery issues typically associated with poorly water and lipid soluble drugs. Conventional size reduction equipment such as media mill and high-pressure homogenizers and formulation approaches such as precipitation, emulsion-solvent evaporation, solvent diffusion and microemulsion techniques can be successfully implemented to prepare and scale-up nanosuspensions. Maintaining the stability in solution as well as in solid state, resuspendability without aggregation are the key factors to be considered for the successful production and scale-up of nanosuspensions. Due to the considerable enhancement of bioavailability, adaptability for surface modification and mucoadhesion for drug targeting have significantly expanded the scope of this novel formulation strategy. The application of nanosuspensions in different drug delivery systems such as oral, ocular, brain, topical, buccal, nasal and transdermal routes are currently undergoing extensive research. Oral drug delivery of nanosuspension with receptor mediated endocytosis has the promising ability to resolve most permeability limited absorption and hepatic first-pass metabolism related issues adversely affecting bioavailability. Advancement of enabling technologies such as nanosuspension can solve many formulation challenges currently faced among protein and peptide-based pharmaceuticals.

## Introduction

**Fig. 1:**
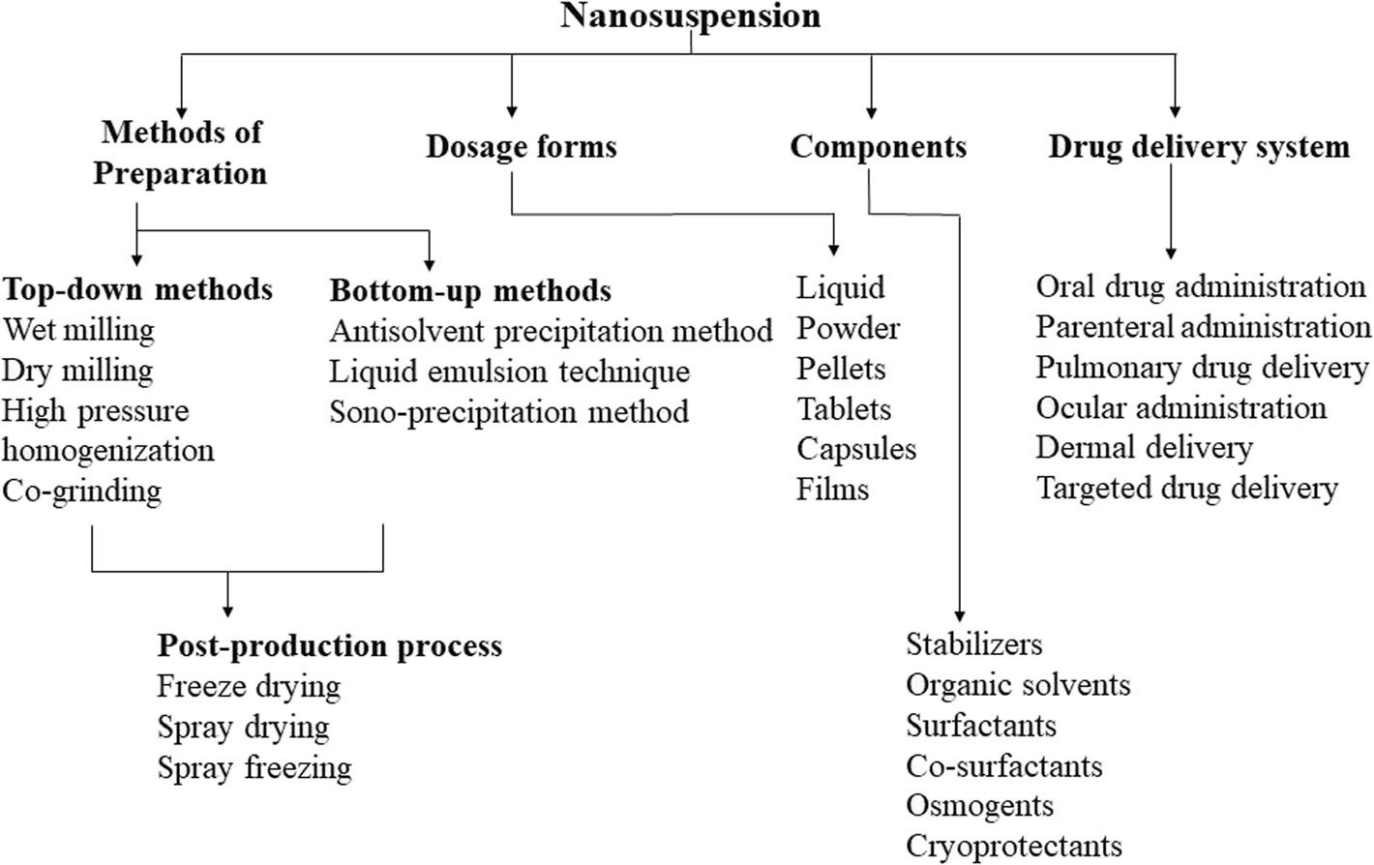
Schematic representation of method of preparation, dosage forms, components and applications of nanosuspensions in drug delivery systems

The advancement in combinatorial chemistry and high throughput screening during the last few decades have generated number of potential drug candidates, which possess excellent target receptor binding. But due to large molecular weight and high log *P* values, these candidates have inherently low aqueous solubility thus restricts further development as a successful dosage form. It is a well-established fact that large surface area offered by particle size reduction can significantly enhance dissolution rate and bioavailability according to classical Noyes-Whitney equation [64]. Pharmaceutical nanosuspensions of drugs are nanosized, heterogeneous aqueous dispersions of insoluble drug particles stabilized by surfactants. In contrast, nanoparticles are either polymeric or lipid colloidal carriers of drugs. Nanosuspension technique is the only option available, when a drug molecule has many disadvantages such as inability to form salt, large molecular weight and dose, high log P and melting point that hinder them in developing suitable formulations. A major limitation of molecular complexation using cyclodextrin in pharmaceutical formulations is their inherent nature to increase the formulation bulk because of large molecular weight of complexing agent [31]. Nanosuspensions can solve such unique drug delivery issues associated with the active pharmaceutical ingredients (API) by retaining it in a crystalline state while enable them with increased drug loading during formulation development. Accommodating large drug amount with minimum dose volume has additional benefits in parenteral and ophthalmic drug delivery system owing to the minimization of excessive use of harmful non-aqueous solvents and extreme pH. Other advantages include increased stability, sustained release of drug, increased efficacy through tissue targeting, minimum first pass metabolism and deep lung deposition. The method of preparation, dosage forms, components and applications of nanosuspensions in drug delivery systems are schematically represented in Fig. 1. Currently, many nanosuspension products of poorly soluble drugs are marketed or under development (Table 1). These advantages have driven towards faster development of nanosuspension technology during last few decades. Despite the intricacies associated manufacturing, selecting appropriate unit operation, equipment and process optimization can counteract these complexities to larger extent.

**Table 1:** Currently marketed pharmaceutical nanosuspension products

Trade name/Company	Drug	Dosage form/Route of administration	Nanosuspension method	Indication
Abraxane®/Abraxia Biosciences	Paclitaxel	Freeze-dried powder for injection/ Parenteral	nab™	Metastatic breast cancer
Cesamet®/Lilly	Nabilone	Capsule/Oral	Coprecipitation	Antiemetic
Emend®/Merck	Aprepiant	Capsule/Oral	Nanocrystal®Elan Nanosystems	Antiemetic
Giris-PEG®/Novartis	Griseofulvin	Tablet/Oral	Coprecipittation	Antifungal
Invega Sustenna®/ Johnson & Johnson	Palperidone palmitate	Liquid nanosuspension/ Parenteral	High pressure homogenization	Schizophrenia
Megace ES®/Par Pharmaceutical Companies	Megestrol-acetate	Liquid nanosuspension/Oral	Nanocrystal®Elan Nanosystems Media milling	Anti-anorexic
Rapammune®/Wyeth	Sirolimus	Tablet/Oral	Nanocrystal®Elan Nanosystems	Immunosuppressant
Tricor®/Abbott	Fenofibrate	Tablet/Oral	Nanocrystal®Elan Nanosystems	Hypercholesterolemia
Triglide®/First Horizon Pharma	Fenofibrate	Tablet/Oral	IDD-P® Skyepharma	Hypercholesterolemia
Avinza®/King Pharmaceuticals	Morphine sulphate	Tablet/Oral	Nanocrystal®Elan Nanosystems	Psychostimulant
Ritalin®/Novartis	Methyl Phenidate HCl	Tablet/Oral	Nanocrystal®Elan Nanosystems	Muscle Relaxant
Zanaflex™/Acorda	Tizanidine HCl	Capsules/Oral	Nanocrystal®Elan Nanosystems	Muscle Relaxant
Focalin®XR/Novartis	Dexmethylphenidate hydrochloride	Tablet/Oral	Nanocrystal®Elan Nanosystems	CNS Stimulant
Ostim®/Heraseus Kulzer EquivaBone®/Zimmer Biomet OsSatura®/IsoTis Orthobiologics NanOss®/Rti Surgical	Hydroxyapatite	Paste/Injection	^a^	Bone substitute
Vitoss®/Stryker	Calcium phosphate	Foam packs, Foam strips/Injection	^a^	Bone substitute
Ryanodex®/Eagle Pharmaceuticals	Dantrolene sodium	Freeze-dried powder for injection/intravenous	^a^	Malignant hypothermia

^a^Not available

The stability of the submicron particles achieved in the nanosuspension is mainly attributed to the uniform particle size, which is formed by different manufacturing techniques. Particles of nanosuspensions must remain unchanged in size throughout its shelf-life otherwise it can initiate spontaneous crystal growth. Therefore, maintaining the uniform particle size distribution can hinders the presence of varying saturation solubility and thereby inhibit any crystal growth due to Oswald ripening effect [66].

### Fundamental principle

Nanoparticles are frequently prepared by reduction/dispersion of large particles to nanosized range like in milling process (Scale down technology) or condensation/aggregation of particles from molecular dispersion to nanosized particles, as in precipitation method (bottom up technique). Since the particle size of nanoparticle is less, it possesses an enormously enhanced surface area compared to its original surface area. Consequently, it can increase saturation solubility determined by Ostwald-Freundlich’s equation as given below:

$$ \log\ \left(\mathrm{S}/{\mathrm{S}}_0\ \right)=\left(2\gamma M/2.303 rT\rho R\right) $$

where *S* is the saturation solubility of small particles of radius (r), S_0_ is the solubility of the large particles (normal solubility), γ is the interfacial tension between solid and liquid, *M* is the molecular weight of the solid, *R* is the gas constant, *T* is the absolute temperature, and ρ is the density of the solid.

The equation is significant when the particle size of the compound is submicron. This makes nanosizing more effective than micronization. Another theory explaining the increased saturation solubility is the formation of high-surface energy surfaces during nanosizing. This disrupt the ideal crystal lattice, thereby exposing the internal hydrophobic surface of the crystal to the aqueous medium. Significant effect of interfacial energy on the saturation solubility between different polymorphic forms of the drug was demonstrated. Similar explanation might be valid true for highly soluble metastable nanosuspension having high surface energy in comparison to more stable coarse suspension that possess low surface free energy and low saturation solubility [36].

The solid drug dissolution rate is also directly proportional to surface area available to dissolution, which can be described by Nernst-Brunner/Noyes-Whitney equation [64];

$$ \mathrm{dx}/\mathrm{dt}=\mathrm{AD}/\mathrm{h}\ \left(\mathrm{Cs}-{\mathrm{X}}_{\mathrm{d}}/\mathrm{V}\right) $$

where, *dx/dt* is the dissolution velocity, *D* is the diffusion coefficient, *A* is the surface area of the particle exposed to the dissolution media, *h* is the diffusion layer thickness, Cs is the saturation solubility of the solute at defined temperature, X_d_ is the concentration of the solute in the media at time, *t* and *V* is the volume of the dissolution media. It is apparent that size reduction to nano-size range will substantially increase dissolution rate due to increase of effective surface area.

A system is said to be thermodynamically stable, if it has low surface free energy (ΔG). The relationship is as follows: ΔG = γ_S/L_ ΔA, where γ_S/L_ is the interfacial tension between solid and liquid. The nanoparticulate system tends to decrease the increased surface area by either preferential solubility of crystal nuclei or regrouping of small particles. Irrespective of the preparation methods, this can cause increase in particle size subsequently results in reduction of surface free energy.

Optimum concentrations of surfactants lower the surface free energy by decreasing the interfacial tension exists between solid and liquid medium. Electrostatic and steric repulsion are facilitated by adding both polymers and ionic surfactants as stabilizers during nanosuspension preparation. Neutral polymers added to the system adsorb onto the particle surface and cause steric repulsion. Further, polymer can inhibit crystal growth and result increase in the particle size. Addition of ionic surfactant to a particle surface previously stabilized with non-ionic polymer allows superior surface coverage than using surfactant alone [95]. Therefore, charged nanoparticles exists in minimum repulsion region of potential energy barrier versus interparticle distance plot. The hydrophobic portion of stabilizers will coat the lyophobic crystal surface and consequently establish stable nanoparticles. Particles in nanosuspension do not settle due to Brownian motion and thereby improves the physical stability. When nanoparticles are introduced into the biological environment, many intermolecular interactions with biological environment can occur resulting in undesired effects such as aggregation, coagulation and precipitation [29]. Therefore, physical characterization tests and methods should be applied to various physical states of nanosuspension as given in Table 2.

**Table 2:** Physical characterization tests and methods

Parameters	Methods
Particle-size distribution (during preparation, transportation and accelerated stability conditions)	Photon correlation spectroscopy, transmission electron microscopy, scanning electron microscopy, atomic force microscopy, scanning tunneling microscopy, or freeze fracture electron microscopy
Surface potential	Laser doppler anemometry, Zeta potential meter
Surface hydrophobicity	Contact angle goniometry, Hydrophobic interaction chromatography
Surface analysis	Static secondary ion mass spectrometry
Crystalline state and amorphous state	X-ray diffraction analysis supported by differential scanning calorimetry scanning electron microscopy, atomic force microscope or transmission electron microscopy
Syringeability and injectability	Texture analyzer
Drainability	Freeness tester
Redispersibility	Powder tester
Solubility	Equilibrium solubility method, Kinetic solubility method
Dissolution	Dissolution test apparatus

### Manufacturing techniques

#### Precipitation techniques

Materials of sub colloidal dimensions are caused to aggregate into particles of colloidal size range. The method involves rapid production of nuclei by preparing supersaturated solution of drug in water miscible organic solvent at optimum temperature and dispersing as small metered amount in non-solvent, water, under rapid stirring [65]. Change in solvent causes high supersaturation conditions, which results in rapid nucleation and at the same time not allowing supersaturation near the nucleating crystals. Rapid nucleation and slow growth rate are the key decisive factors for successful thermodynamically stable crystal form according to classical Ostwald law of nucleation [79]. Generation of finely dispersed uniform sized drugs, simple and economical process and ease of scale-up are the main advantages of precipitation method.

High supersaturation condition can create acicular or needle like crystal habit which can be broken down quite easily to generate additional nuclei formation at the expense of crystal growth. Secondly, non-introduction of impurities can cause imperfections or defects in crystal lattice that can be homogenized to decrease the particle to nanometer range. Crystal growth must be suppressed by appropriate concentration of surfactant or selective crystallization inhibitors. A patented technology (Lucarotin® (BASF) based on precipitation method to produce amorphous drug nanoparticles is utilized for pharmaceuticals is named as Nanomorph™ (Soligs/ Abbott/ Patent No. D 19637517),

#### Homogenization

In homogenization, size reduction of the particles is accomplished by driving suspension under high pressure (100–1000 bars) through a valve that has a small aperture [49]. The static pressure reduction due to sudden drop in fluid velocity causes cavitation induced implosion forces and shock waves in the liquid medium break down the microparticles (< 25 μm) to nano-size range. Further, shear forces due to the collision of particles and high velocity helps to fracture particles with inherent crystal defects. Viscosity enhancers can increase particle density within dispersion region, inhibit crystal growth hence improves the process of nanosizing [10]. Homogenization can transform metastable amorphous particle prepared by the precipitation method to stable crystal form. Generally, multiple cycles are required to produce particles of desired size ranges. Ease of scale-up, adaptability of the process to dilute or concentrate suspensions, low risk of contamination and feasibility for aseptic manufacturing are the key benefits of this method. The drawbacks of the techniques are pre-requirement for micronized particles, repeated cycles, high energy technique and possibility of contamination that could occur from the metal wall of the container.

Many homogenization technologies has been either patented or patent application are pending such as Hydrosol (Novartis/ Patent No. GB 2269536), Nanocrystal™ (Elan Nanosystems/ Patent No. US 5145684), Dissocubes® (Skye Pharma/ Patent No. US 5858410), Nanopure (Pharma Sol/ Patent Application No. PCT/EP00/0635), NANOEDGE™ (Baxter/ Patent No. US 6884436).

#### Wet milling or media milling

In this method, nanosuspension are prepared by high shear ball mill or media mill. The size reduction within the chamber charged with drug, stabilizer (s) and water is carried out by both impact and attrition of particles. Low energy utilization, ease of scale-up, minimum batch to batch variation and capability to handle large quantities of material, and four approved Food and Drug Administration (FDA) drugs makes this process more attractive. The amount of material in the mill is of considerable importance since too much feed produces a cushioning effect and too little causes loss of efficiency and abrasive wear of the mill parts. The wear and tear of milling media may occasionally introduce residues in the finished product. However, this problem has been minimized by using highly crosslinked polystyrene resin milling medium. Extended milling process time can introduce more amorphous fraction into the materials, which may lead to instability. Wet-milling process to manufacture uniform sized nanosuspension using small hard zirconium dioxide beads have been described [63]. The X-ray diffraction analysis showed that the initial crystal nature as well as crystallinity of the active remained same even after the wet-milling process.

#### Dry co-grinding

Stable nanosuspension prepared by dry co-grinding technique using various polymers and copolymers such as polyvinyl pyrrolidone, hydroxypropyl methylcellulose (HPMC), polyethylene glycol, sodium dodecyl sulphate and cyclodextrin derivatives [88] have been described. Unlike wet grinding process, dry grinding methods are more economical and can be carried out without addition of any toxic solvents. The most significant effect of this method is the improvement of surface polarity and modification of major portion of crystalline state of the drug to amorphous form. Controlled stability of the amorphous phase could significantly improve the saturation solubility and hence dissolution rate of poorly soluble drug nanosuspensions [86].

#### Emulsion-solvent evaporation technique

An emulsion is formed by first dissolving drug in an organic solvent or cosolvents and subsequently dispersing in an aqueous phase containing a surfactant, which act as a stabilizer. Rapid evaporation of solvent under reduced pressure instantaneously produce nanosuspension. Key factors that should be considered during emulsification method are globule size and concentration of stabilizer.

#### Ultrasound assisted sonocrystallization method

It is a novel approach used for the preparation of stable nanosuspension. Ultrasound employed at the frequency range of 20–100 khz enhances particle size reduction and controls the size distribution of pharmaceutical active ingredient. It is also considered as an effective technique for minimizing the nucleation and crystallization process [80].

#### Miscellaneous methods

Nanosuspension of the drug can also be achieved by dilution of emulsion, thereby causing full diffusion of dispersed phase into the continuous phase resulting in the production of nanosuspension. Microemulsion can be treated in similar manner for the production of nanosuspensions. The effect of globule size and amount of surfactant (s) on the drug uptake of internal phase should be examined to produce optimum drug loading. Nanosuspension developed by such methods must be cleared from adhering solvents and other ingredients by means of ultrafiltration technique to make it convenient for administration. Lyophilization of the nanosuspensions shall be done to improve the physical and chemical stability and to overcome the incompatibilities between the various formulation components. Sterilization of the nanosuspensions can be done either by membrane filtration (< 0.22 μm), steam heat sterilization or gamma irradiation. Literature suggests that optimization of bottom up nanosuspension approach requires appropriate selection and setting suitable concentration of excipients such as surfactant and polymer [79].

#### Formulation components

The most frequently used excipients in nanosuspensions are stabilizers, polymers, surfactants, osmotic agents, organic solvents, cryoprotectants, buffers, complexing agent, buffers, organoleptic agents and preservatives.

##### Stabilizers

It was reported in the literature that APIs carrying high log P and high enthalpy values have more practicability of developing a stable nanosuspension by both steric and electrostatic stabilization [19]. Further, physical properties such as molecular weight did not demonstrate to have a direct influence on the particle size or stabilization step. The zeta potential (ζ) measured at the shear plane determines the degree of repulsion between similarly charged adjacent particles in the system. Though, the magnitude of zeta potential typically signifies the physical stability of the nanosuspensions, it may not be a good indicator for stability achieved through electrostatic stabilization. This is because electrical properties at the interface is also due to the dissociation of charged functional groups on the particle surface influenced by both pH of the medium and pKa of the drug. The dynamic state of nanosuspension formation is a complex interaction involving various factors particularly surface free energy and functional groups. Faster surface adsorption rate of stabilizer in comparison to newly created surface during various preparation methods may dictates the stability of the nanosuspensions as well as efficiency of the selected technique.

The polymorphic and amorphous-crystalline transformation in nanosuspension can significantly change the solubility and clinical effect. Solid state stability involving crystal defects of API during milling process, percentage crystalline/amorphous content can be studied in comparison with physical mixtures by powder X-Ray diffractometer (XRD) supported by other physical characterization methods [25]. In contrast to XRD, small angle X-ray scattering can provide information about the size, shape, orientation, and crystalline and amorphous state of a variety of polymers, proteins, and nanomaterial bioconjugate systems in solution [51]. Thermal characteristics of the air/freeze dried nanosuspension powder is typically evaluated using modulated differential scanning calorimetry (Table 2). Maintaining the in vivo stability is critical to establish long-circulating characteristics and to achieve passive drug targeting through enhanced permeation and retention (EPR) effect. Since nanosuspensions are frequently prepared with aqueous medium, common chemical stability issues such as oxidation and hydrolysis must be addressed. Thus, the important function of stabilizer to prevent agglomeration or aggregation due to high surface energy of the nanoparticles. Any variation in particle size distribution and polydispersity index at different stages of nanosuspension such as during production, storage and stability conditions can be examined using various types of particle size analyzers technique (Table 2). Other key roles of stabilizer are wetting of hydrophobic drug particles, prevent Ostwald’s ripening and provide steric or ionic repulsion to make physically stable product. The concentration of stabilizer has significant impact on the physical stability and in vivo performance of nanosuspensions. Various types of stabilizers have been investigated such as cellulose derivatives, phospholipids, poloxamers, non-ionic surfactants and polyvinylpyrrolidone.

##### Poloxamers

These synthetic polymers are generally regarded as safe (GRAS) by FDA for oral, parenteral and topical pharmaceutical applications. Most frequently used Poloxamer in nanosuspension is Poloxamer 188 and Poloxamer 407. Hydrophilic-lipophilic ratio, morphology, functional groups and molecular weight are the key factors, which determine the crystal growth and stability of nanosuspension [47].

##### Amino acid-based stabilizers

Leucine copolymers have been demonstrated to successfully produce stable drug nanocrystals in aqueous medium [45]. Lecithin is preferred as stabilizing agent for sterile, steam heat sterilizable parenteral nanosuspensions. Albumin has been employed as a surface stabilization and drug targeting at various concentrations as low as 0.003% up to 5% in nanosuspension [92]. Other pharmaceutically acceptable amino acid co-polymers used for the physical stability of nanocrystals were arginine, proline, and transferrin.

##### Cellulose based derivatives

HPMC, hydroxypropyl cellulose (HPC), hydroxyethyl cellulose (HEC) have been widely used as stabilizing agent in nanosuspensions. The underlying mechanism of steric stabilization provided by these polymers is due to surface adsorbed hydrophobic groups [56].

##### D-α-Tocopheryl polyethylene glycol 1000 succinate (Vitamin E TPGS)

Vitamin E polyethylene glycol succinate is an esterified, water soluble vitamin E (tocopherol) derivative used in many nanosuspension formulation as a stabilizing and solubilizing agent [21]. High physical stability, and low toxicity profile consider it as a most effective excipient for oral, ophthalmic and parenteral applications.

##### Miscellaneous

Soluplus® is a novel excipient made of polyvinyl caprolactam-polyvinyl acetate-PEG copolymer developed by BASF industries. It has been used as a stabilizer in many nanosuspension with enhanced stability and increased dissolution rate and bioavailability [74]. Water soluble polymers such as polyvinyl alcohol (PVA), polyvinyl pyrrolidone, and PEGylated chitosan used as stabilizers was found to significantly increase the dissolution rate and bioavailability of nanosuspensions. Functionalized surface coating on low soluble drug, beclomethasone dipropionate was carried out with hydrophobin, a protein-based surfactant. Adaptability for surface modification by genetic engineering make it suitable for different drug delivery applications [81].

##### Surfactant and co-surfactants

The selection of surfactant and co-surfactant are important, when nanosuspensions are prepared using microemulsions as template. It can influence the phase behavior, solubility of drug as well as drug loading in the internal phase. Different types of surfactants such as Tweens [37], sodium dodecyl sulphate [78] and co-surfactants such as bile salts, transcutol, glycofurol, ethyl alcohol and isopropyl alcohol were successfully used in the stabilization and development of nanosuspensions.

##### Organic solvents

Class three organic solvents with less toxic potential to humans such as ethyl alcohol, acetone, butanol, ethyl formate, ethyl acetate, ethyl ether, methyl acetate, methyl ethyl ketone, triacetin are preferred over conventional hazardous residual solvents. Water miscible organic solvents can be used as internal phase to solubilize drug substance, when nanosuspensions are prepared based on emulsion solvent evaporation technique.

##### Other ingredients

Complexing agent such as cyclodextrin derivatives have been explored for the improvement of dissolution and bioavailability of actives in nanosuspensions [31]. Nanosuspensions like coarse suspension might contain buffers, preservatives, osmotic agents, cryoprotectants, organoleptic agents, depending on the type, route and physicochemical nature of drug.

### Nanosuspensions in drug delivery systems

#### Oral drug delivery

Oral suspension ensures chemical stability while allowing liquid medication, which is preferred in geriatric and pediatric age groups. Other advantages are masking the bitterness of drugs, extending the duration of action of drugs, improving the aqueous solubility of poorly water-soluble drugs, dissolution and bioavailability enhancement of drugs. Further, if the drug is insoluble in water and other solvents are not allowed, then suspension is the preferred choice.

Nanosizing of drug can cause significant improvement in dissolution rate because of large increment of surface area and subsequent rise in saturation solubility. This leads to an increased dissolution velocity and concentration gradient across gastrointestinal tract causing increased absorption and significant enhancement of bioavailability. Thus, nanosuspensions are most beneficial for drug candidates belongs to class II and class IV as per the Biopharmaceutical Classification System (BCS). The bioavailability studies of poorly water soluble drug, fenofibrate formulated as nanosupension type, Dissocube® showed two-fold enhancement in terms of rate and extent compared to the reference formulation of micronized fenofibrate suspension [23].

The in vivo test of nitrendipine nanosuspension (~ 290 nm) in rats showed that the maximum concentration of drug (*C*_max_) and area under plasma-drug concentration time profile (AUC) were approximately 6 folds higher in comparison to marketed tablets [89]. Pharmacokinetic study demonstrated significant improvement in the oral bioavailability in rabbits by naringenin nanosuspension in comparison to naringenin solution [70]. Pharmacokinetic evaluation of fluvastatin nanosuspensions in rats showed 2.4 folds improvement in bioavailability in comparison to fluvastatin capsule control group [48]. Faster absorption from naproxen nanosuspension decreased time to reach maximum concentration (T_max_) by nearly 50% and increased AUC to about 5 folds, when compared to conventional tablet and suspension [55]. The *C*_max_ and *T*_max_ of the prepared nanosuspension incorporated mucoadhesive buccal films of carvedilol was increased due to both enhanced surface area and by avoiding hepatic metabolism [71].

Selection and optimizing the concentration of stabilizers are important for the successful formulation and stabilization of nanosuspension. A combination of co-processed nanocrystalline cellulose-carboxy methyl cellulose was successfully used as a steric stabilizer in the preparation of baicalin nanosuspension [90]. Similarly, spray dried nanosuspension of itraconazole for bioavailability enhancement was stabilized by including poloxamer 407 or low viscosity (50 cp) HPMC. In vivo studies in rats revealed that AUC_0–36_ calculated from dried itraconazole nanosuspensions was significantly higher (~ 2 folds) than the commercial Sporanox® beads in both fed and fasted conditions (*p* < 0.05).

Though, particle size reduction increases dissolution rate, enormous surface free energy possessed by nanoparticles can sometimes results in reduced uptake of drug. The aqueous solubility of the drug was enhanced with considerable improvement of bioavailability was exhibited after oral administration of amphotericin B nanosuspension [35]. Large surface area of nanoparticles can also lead to mucoadhesion, which can prolong gastrointestinal residence time and improve bioavailability. But the penetration to underlying mucosa can be affected by both surface charge and the nature of the surfactant [4]. Transcytosis of nanoparticles is adversely affected by the enteric coating after the initial adsorption by salivary proteins [17]. Nanoparticle uptake by rat gastrointestinal mucosa indicated that M-Cells in Peyers patches involves in lymphatic transport of drug to systemic circulation bypassing first pass metabolism by liver. This mechanism is beneficial for targeting lymphatic diseases or lymph mediated diseases. Lower inter-individual variability indicated that nanosuspension enhance the absorption from the targeted site of small intestine irrespective of food intake [60]. Despite the promising advantages of nanosuspensions, it is not applicable in situations, where bioavailability is compromised by biotransformation and/or permeation related problems particularly observed in class II and class IV drugs. Nanoengineering these crystals using various agent may minimize both permeation [3] and gut related metabolic issues [40].

Nanosuspensions offer potential advantages such as reduction of dose as well as cost of therapy, avoiding dose dumping in the body, minimizing fast/fed state plasma level fluctuation and intersubject variability. Sustained release technology has evolved remarkably during last several decades, but it is restricted to few hydrophobic drugs. Nanocrystals in nanosuspension have the capability to incorporate potentially all hydrophobic drugs in various sustained release methodologies. In addition, drug nanoparticles can be included in tablets, capsules and pellets using conventional manufacturing method.

### Modification of liquid nanosuspensions to solid intermediate

#### Powders

Powders are the starting process material for most solid dosage forms such as granules, tablets and capsules. Nanosuspensions of poorly soluble drugs are often converted into different powder dosage forms for oral administration as shown in Table 3. Nanosuspensions can be converted to dry powders either by freeze drying, spray granulation, vacuum drying, or spray drying techniques. The key challenge is in preserving the redispersibility of nanoparticles upon reconstitution of powder form with water or in gastric fluids. Powders are both the simplest dosage forms and the basis of many other solid dosage forms, such as tablets, capsules, and so on. Many drugs or ingredients are also in powder form before processing. A good redispersants should rapidly disperse agglomerates formed during the drying so that the original particle size is regained within a short span of time. Redispersants must be added previously to the nanosuspensions before drying step. Typical redispersants used are sucrose, trehalose, maltodextrin, lactose and mannitol, which is also used as cryoprotectant during lyophilization. In vivo pharmacokinetic evaluation of ritonavir nanosuspension in rats demonstrated a significant rise of C_max_ and AUC_0 − t_ values, when compared to coarse powder and marketed product (Norvir®) evaluated among fed group human volunteers [27].

**Table 3:** Nanosuspensions modified as powder dosage forms for oral administration

Types of powder dosage forms	Drug	Conversion method	Comment	References
Oral Powders	Cefixime	Cospraying with carrier, sorbitol	Solubility of cefixime is 5 fold than coarse powder	[2]
Oral Powders	Itraconazole	Freeze drying with microcrystalline cellulose	Faster dissolution with increasing amount of MCC	[75]
Oral Powders	Naproxen, Novartis Compound A	Spray granulation with mannitol	Top spray yielded finer, redispersable particles; No differences in AUC	[16]
Oral Powders	Rutin	Freeze drying and Spray drying sodium tripolyphosphate and Chitosan	Significant increase in particle size by both methods	[68]
Inhalable Powders	Ibuprofen	Spray drying with mannitol and/or leucine	Fine particle fraction dependent on both the leucine and mannitol to drug ratio (*p* < 0.05)	[52]
Inhalable Powders	Niclosamide	Spray drying with mannitol	Strong quorum sensing inhibiting activity against *Pseudomonas aeruginosa*	[12]

#### Pellets

As a multiparticulate dosage forms, pellets offer number of advantages such as controlled release of drugs, release independent of gastric emptying rate, less chance of dose dumping, minimum local irritation, adaptability to accommodate different release profile, ability to combine different drugs and patient compliance.

Dried indomethacin nanosuspensions were prepared by spraying the nanosuspensions onto pellets by fluid bed coating method. Similar dissolution profiles were demonstrated between dried pellets with nanosuspension and drug nanosuspensions [24]. Pellets incorporating nanosuspension of ketoprofen for sustained release of drugs up to 24 h duration has been disclosed [82]. Mucoadhesive hydrocortisone nanosuspension was produced by high pressure homogenization method. Pellets were initially spray coated with nanosuspensions and further coated with enteric polymers to maintain a controlled drug release. In vitro dissolution studies demonstrated an enhanced dissolution rate and drug release from the nanocrystals present in the pellets [59]. It was suggested that spray coating of nanosuspension containing water soluble binder to pellets followed by enteric coating is most likely to control the release rate for high dose drugs. Similarly, pellets prepared by spheronization-extrusion technology with same methodology to make matrix cores can be applied for low dose drugs [58].

#### Tablets

Pharmaceutical tablets are solid unit dosage form containing drug substances usually prepared with the aid of suitable pharmaceutical excipients by compression. In most of the investigations, granules were prepared by either freeze drying or spray drying techniques. Nanosuspension after conversion to dry powder by these methods can be compressed as tablets by molding or compression. Nanosuspension can also be converted to tablet form by directly freeze drying the nanosuspension in a blister pack. Since the stresses during freezing and drying cycles are diverse, multiple stabilizers such as sugars, sugar alcohols, polymers and amino acid are often used to contribute adequate protection and stability which are essential for the drug (s). These excipients are frequently combined to build either amorphous or crystalline structure of freeze dried nanosuspension.

Unlike in case of electrostatic stabilization, steric stabilizers are comparatively unaffected in presence of electrolytes and other excipients added during the formulation of tablets. Thus, the selection of appropriate steric stabilizers based on the properties of API can produce a stable nanosuspension at varying pH of gastrointestinal tract after oral administration. A zeta potential value of around − 20 mV can be considered as ideal for a nanosuspension system stabilized by both steric and electrostatic methods [85]. Nanocrystals in a tablet above certain limit can aggregate to a larger extent under the compressive force during tableting process. This can decrease the dissolution velocity and bioavailability particularly poorly water soluble BCS II drugs. Formulation of low dose drugs with total nanoparticle content less than 1% relative to total tablet weight is effective in releasing it as fine nanosuspension to the solution [9].

Naproxen granules were prepared from nanodispersion by spray drying technique and tablets were prepared by compressing the same with a bulking and stabilizing agent, mannitol and a disintegrating agent. It was found that the dissolution of the nanodispersion was finished within 1 min under sink and non-sink conditions [7]. Spray drying technology has also been used to prepare nanocrystals of lovastatin, a poorly soluble drug from nanosuspension using 20% polyvinyl pyrrolidone K17 and 5% sodium lauryl sulphate as stabilizers. Optimized sustained release tablets were prepared using lactose (diluent), Avicel PH101 (compressing agent) and Ac-Di-Sol (superdisintegrant) [93]. Increased dissolution has been reported with oral nanosuspension tablets of nebivolol hydrochloride, a poorly water-soluble drug. Enhanced dissolution rate is due to the transformation of crystalline form of the drug to amorphous state, which was later proved by X-ray diffraction studies. In a similar study, orally disintegrating tablets piroxicam tablets were prepared using nanosuspensions to which poloxamer 188 was used as stabilizer, showed a superior dissolution rate compared with the orally disintegrating tablets prepared from coarse piroxicam [42]. Increased dissolution rate is owing to increased surface area associated with nanosized drug particles. In another study, spray dried nanosuspension of risperidone orally disintegrating tablets showed improved dissolution rate in comparison to marketed orally disintegrating tablets [62]. In a continuation of the study, the effect of different excipients on the piroxicam dissolution properties was investigated. It was established that gelatin or croscarmellose as excipient exhibited a faster piroxicam dissolution rate, when compared with the marketed formulation and orally disintegrating tablets formulated using xanthan gum. This study additionally implies the significance of different excipients used in the formulation.

Including polymers like Poly (DL-lactide-*co*-glycolide) and complexing agent such as cyclodextrin are a well-known approach to enhance the biopharmaceutical performance of poorly soluble drugs. It was confirmed that concentration of nanosuspension, spraying rate and atomization air pressures are the key factors that influence the various physicochemical properties of granules such as redispersibility and particle size distribution [28].

#### Capsules

Novartis compound A and itraconazole nanosuspension dried as powders and subsequently filled in capsules was found to enhance bioavailability after oral administration in beagle dogs [6] and rats [39], respectively. In vitro dissolution test and in vivo studies in rats demonstrated a marked improvement in bioavailability of glimepiride nanocrystal-loaded capsules compared to the marketed formulation [15].

#### Oral films

Oral films or orodispersible films have many advantages than other oral dosage forms since it undergoes quick disintegration and dissolution in the oral cavity, rapidly delivers the drug across oral mucosa bypassing hepatic metabolism and resulting enhancement of bioavailability.

Buspirone fast dissolving oral films was prepared from nanosuspension by solvent evaporation method using film forming agents HPMCE5 and PVA. Buspirone oral film showed excellent physicomechanical characteristics, good stability and in vitro studies exhibited burst release followed by sustained drug release [5]. It was anticipated that incorporating nanoparticles in fast dissolving oral films can increase both dissolution and permeability characteristics of many poorly aqueous soluble drugs. Fast dissolving oral films incorporating nanoparticles of lercanidipine, a poorly aqueous soluble and low bioavailable drug were prepared via antisolvent evaporation method. Significant enhancement of i*n vitro* dissolution rate and ex vivo steady state flux was noticed from the formulations [11].

Feasibility studies of low bioavailable, quercetin fast dissolving oral films prepared using maltodextrins as film forming material and glycerin as plasticizer indicated that the inclusion of nanocrystals did not influence the elasticity and ductility. The dissolution rate was found to be better than that of bulk drug [41]. Pharmacokinetic studies of lutein nanocrystals fast dissolving oral films in rats indicated a major reduction of T_max_ and considerable increase of C_max_ compared to oral solution. Further, the AUC_0-24h_ of nanocrystal fast dissolving oral films was ~ 2-folds larger than that of the oral solution thereby confirming the drastic improvement of both rate and extent of bioavailability [50].

Recently, nanosuspension based mucoadhesive film was prepared with carvedilol nanosuspension containing layer held between mucoadhesive and backing layers. Nanosuspension was incorporated into hydrogel prepared from HPMC and Carbopol 934P using PEG400 as plasticizer. In vivo studies performed in rabbits displayed significant rise in the relative bioavailability, when compared to commercial tablets [71].

#### Parenteral drug delivery

Intravenous and intraspinal preparations are seldom given in a form other than aqueous solutions. The danger of capillary blockage, particularly in the brain deter the use of other forms via intravenous administration. Though, microemulsions have been used such as total parenteral nutrition, particle size of the dispersed phase is rigidly controlled below 5 μm. Parenteral products can be given as solutions, suspensions, or emulsions through either intramuscular, subcutaneous or transdermal route of administration.

Injections of poorly aqueous soluble is challenging and often formulated with cosolvents, solubilizing agent, selecting suitable salt forms and complexing agents such as cyclodextrin. Due to the limitation associated with the excessive use of toxic cosolvents, solubilizing agent and complexing agents, these techniques suffer from lack of solubilizing power and parenteral acceptability. In this context, nanosuspension can be best alternative in such conditions since all hydrophobic drugs can be nanosized while circumvent all the problems frequently encountered during formulation of parenteral products. Improvement in bioavailability facilitate dose reduction, cost effectiveness of therapy without affecting therapeutic outcome of the drug.

Safety profile of the paclitaxel nanosuspension was increased many folds higher than the commercial taxol injection, which employs cosolvent mixtures to solubilize the drug. Paclitaxel nanosuspension resulted no death at maximum dose of 100 mg/kg whereas taxol at dose of 30 mg/kg demonstrated a death rate of 22% in human lung xenograft murine tumor. Therapeutic efficacy of paclitaxel nanosuspension was enhanced in comparison to taxol employing transplantable mouse 16/c mammary adenocarcinoma as a model [55].

Cytotoxicity studies indicated that superior cytotoxicity in Hela and MCF-7 cells with curcumin nanosuspension than curcumin solution. Small particle size (~ 250 nm) has increased dissolution rate and retention of crystalline nature improved physical stability of curcumin. Further, the safety evaluation studies with nanosuspension demonstrated minimum local irritation, allergic reaction, phlebitis and decreased rate of red blood cell lysis [18].

Improvement in stability and efficacy was noticed with aphidicolin, a low aqueous soluble antiparasitic drug [34] and hydrophobic antileprotic drug, clofazimine, when formulated as nanosuspension [67].

It is important to note that soon after parenteral intake, nanosuspension undergoes opsonization and phagocytosis due to uptake by Kupffer-Bowcisz cells located in liver [57]. The natural uptake by these cells serve as reservoir or depot and thereby control or prolong the duration of action of the drug [76]. Targeting to macrophages using antibiotic nanosuspension is beneficial since many pathogens subvert the process of phagocytosis and replicate inside macrophages. In contrast, various research investigations are in progress to avoid uptake by macrophages by varying the size, shape and surface properties of nanosuspensions.

Surface properties of the nanosuspension can be modulated in order to change the protein adsorption patter. Factors like physicochemical characteristics of the drug particles, dose, duration of administration, drug-protein binding properties, solubility in body fluid pH affect the biodistribution and pharmacokinetic evaluation of the nanosuspension after parenteral administration.

Pharmaceutical composition for the intravenous administration of sparingly soluble staurosporine derivative N-benzoyl- staurosporine nanaosupension having particle size 5–20 nm has been patented [87]. The key excipients included in the composition are polyoxyethylene/ polyoxypropylene block copolymer, Poloxamer 188, phospholipid surfactant, lecithin, water soluble osmotic agent, mannitol and co-solvents, water and ethanol.

Sustained release natural progesterone nanosuspension was developed and then successfully dispersed in a thermosensitive gel matrix. It was demonstrated that sustained release action after intramuscular injection in rats was continued up to 36 h and thus anticipated to provide a much safer alternative for semi-synthetic progesterone [73].

Safety and efficacy of long acting GSK1265744 (744) and rilpivirine (TMC278) nanosuspension were evaluated after multiple intramuscular dosing in healthy subjects. All volunteers achieved the steady state plasma concentration within 3 days and clinical data proved the potential use of long acting 744 and rilpivirine injection in HIV-1 treatment [77].

Significant reduction in the viability of undifferentiated/anaplastic thyroid cancer cell line was demonstrated, when tested with docetaxel (100 and 1000 μM/ml) loaded nanoparticles stabilized by 5% sodium deoxycholate [30].

#### Ophthalmic drug delivery

Low ocular bioavailability of many drugs from conventional ophthalmic drug delivery system is largely due to the major anatomical, physiological and physicochemical barriers of the eye. Nanosuspensions offer a means of administering increased concentrations of poorly soluble drugs and extended residence time to targeted site of cul-de-sac. Nanosuspension can address many problems of conventional suspensions such as poor intrinsic and saturation solubility in lachrymal fluids, low ocular bioavailability and irritation due to the large particle size. Further, it can avoid high osmolarity generated by ophthalmic solution dosage forms. Nanosuspension due to its peculiar nature, can improve the saturation and inherent solubility of hydrophobic drugs in lachrymal fluids while allowing sustained release and prolonged residence time in cul-de-sac.

Poor ocular bioavailability of many drugs from conventional eye drops is chiefly due to the physiological barriers of the eye. Investigations of poly (lactic-co-glycolic acid) based sparfloxacin ophthalmic nanosuspension demonstrated an improvement in precorneal retention time and ocular permeation. In addition, the developed lyophilized nanosuspension was found to be more stable than conventional marketed formulations [22]. Nanosuspensions shall be dispersed further in selected ointment, hydrogel or mucoadhesive base to control the release and residence time depends on the physicochemical nature of the drug.

Long term disease management of fungal infections in the eye is a challenging task because of the incapability of the delivery system to provide adequate drug distribution without affecting intraocular structures and/or systemic drug exposure. In vitro release studies of antifungal drug, amphotericin loaded Eudragit RS-100 nanosuspension (150–290 nm) is proposed as an efficient vehicle for delivery for 24 h and no ocular irritation was observed after topical instillation into rabbit’s eye [13]. Poly (methyl methacrylate) polymers like Eudragit® RS100 based nanosuspensions containing piroxicam has been successfully tested for ocular delivery in endotoxin-induced uveitis [1] and Eudragit RL100 polymeric nanosuspension loaded with sulfacetamide was successfully evaluated for ocular delivery [53]. Polymethacrylate polymer such as Eudragit RS 100 and RL 100 was used for the preparation of ophthalmic nanosuspensions of flurbiprofen and ibuprofen [8]. Nanosuspensions were evaluated for drug content, particle surface charge, particle size distribution, in vitro drug release and ocular irritation. Comparison between commercial ophthalmic suspensions demonstrated far superior in vivo performance of prepared ophthalmic nanosuspension.

Application of conventional dosage forms such as ointment, solutions and suspension are limited due to poor ocular bioavailability and different anatomical and pathophysiological barriers existing in the eye. Recent developments and findings of various nanoparticulate systems like nanosuspensions can be appropriately exploited to apply in ocular drug delivery system.

#### Pulmonary drug delivery

Potentially, nanosuspensions can minimize many problems associated with conventional dry powder inhalers and suspension type inhalation aerosols. Conventional aerosols have many limitations such as limited diffusion and dissolution in the alveolar fluids, rapid clearance and short residence time due to ciliary movement, deposition in pharynx and upper respiratory tract due to agglomeration and aggregation of the particles [32]. Nanoparticulate nature of the drug can offer quick onset of action due to rapid diffusion and dissolution in the alveolar fluids. Furthermore, it can sustain the release of drug because of its increased affinity to the mucosal surfaces. Antioxidant coenzyme Q10 nanosuspension stabilized with PEG32 stearate demonstrated maximum respirable fraction (70.6%) having smallest mass median aerodynamic diameter (3.02 μm) in comparison to nanosuspension stabilized with lecithin and Vitamin E TPGS. In vitro cellular toxicity carried out utilizing A549 human lung cells showed no noticeable cytotoxicity for the nanosuspension [72]. Due to the unique physicochemical characteristics, uniform and narrow size distribution of the nanoparticles, it is unlikely to cause uneven drug distribution and drug delivery in lung, when compared to microparticulate inhalation aerosols. The lung distribution rate of hydrophobic budenoside nanosuspension was found to be very high (872.9 ng/g) and exceptionally different (*p* < 0.05), when compared to coarse drug particles (*p* < 0.01) and micronized drug particles [94].

Conversion of nanosuspensions to solid oral and inhalable dosage forms lessens the physical instability associated with their liquid state and enables targeted drug delivery. Most frequently used solidification methods include spray and freeze- drying techniques. Redispersibility of solid nanocrystalline formulations is essential for potential oral and pulmonary clinical application [52].

#### Targeted drug delivery

Nanosuspensions have the potential capacity to investigate as targeted drug delivery system by surface modification in order to circumvent the phagocytosis by macrophages. Active or passive targeting using stealth nanosuspensions using various surface coatings is an interesting area to be explored. Significant difference in the efficacy of buparvaquone, an antiparasitic nanosuspension has been noticed, when administered in presence or absence of mucoadhesive polymer [61]. Because of the prolonged accumulation of drug at the targeted site, drastic decline in the infectivity episode was exhibited by nanosuspension with mucoadhesive polymers. A comparative bioadhesion study was carried out between micro and nano carriers to the mucosa of colon [44]. It was found that nanosized carriers has the potential capability for targeted delivery of drugs to the inflamed colonic mucosa case of inflammatory bowel disease. Studies carried out with budenoside nanosuspension coated with Pluronic F-127 showed significant reduction of local colorectal tissue inflammation as confirmed by decreased inflammatory macrophages and IL-β producing CD11b + cells [14]. Recently, mucus permeating budenoside nanosuspension enema for targeted delivery to inflammatory bowel disease was developed [14]. Mucosal penetration was aided by surface coating with water soluble or hydrophilic polymers, most typically polyalkylene oxide polymers or copolymers such as Poloxamer F127.

Pulmonary targeting of amphoteric B as nanosuspension is reported to be more effective than stealth liposome in conditions of aspergillosis [38]. Most probably, similar strategy using nanosuspensions can be utilized to target gastrointestinal sites such as duodenum, colon, rectum that are susceptible to bacterial infection such as Helicobacter pylori or fungal infections due to *Candida albicans*. Two types of biodegradable, lipid based nanosuspensions was developed, a poly (ethylene glycol)- modified docetaxel-lipid-based-nanosuspensions to enhance the cycle time of the drug inside the tumor site and docetaxel-lipid-based-nanosuspensions employing folate as the target ligand. Data from the in vivo antitumor efficacy and biodistribution studies indicated that docetaxel-lipid-based-nanosuspensions demonstrated higher antitumor efficacy significantly by subsiding the tumor volume (*P* < 0.01) as a result of increased residence time of the drug within the tumor [84].

Though, nanosuspension technology is undergoing rapid expansion during last few decades, the in vivo *safety* and health implications of nanosuspension should be seriously considered during development and scale-up [83]. Nanosuspension has the potential ability to cross blood-brain barrier due to nanosize range and hydrophobicity of the drug. Donepezil loaded nanosuspension as a potential brain targeted drug delivery system for Alzheimer’s disease was tested in Sprague–Dawley rats. The nanosuspension with particle size ranges between 150 and 200 nm was administered to brain via intranasal administration. The C_max_ showed significant (*p* < 0.05) concentration of donepezil in brain (147.54 ± 25.08 ng/ml) in comparison to suspension (7.2 ± 0.86 ng/ml), at an equal dose of 0.5 mg/ml [54]. In another study, amphotericin B nanosuspension was developed as a brain delivery system. Results indicate that nanosuspensions coated with Tween-80 and sodium cholate enhanced penetration to brain and remarkably inhibited encephalitis causing amoeba, *Balamuthia mandrillaris,* in vitro, however showed low in vivo inhibitory activity [46].

Targeted drug delivery into the vaginal tract to prevent pre-term birth rate exploiting mucoinert progesterone nanosuspension was developed. Characteristic plasma progesterone double peak noticed in humans was also observed in pregnant mice after vaginal administration thus confirming uterine recirculation [26]. Passive targeting through PEGylation and coating with polysorbate-80 can cause deposition of apolipoprotein E on the polymeric nanoparticles, which will promote brain uptake by endothelial cells.

#### Transdermal drug delivery

Diclofenac sodium, a non-steroidal anti-inflammatory drug was successfully dispersed into isopropyl as a nanosized solid-in-liquid suspension via complex formation using surfactant, sucrose erucate. The resultant formulation enhanced the steady state flux of the drug up to 3.8-fold when compared with the control in Yucatan micropig skin model. The size of the nanosuspension was found to depend on the weight ratio of the surfactant and the average diameter of the nanoparticles was 14.4 nm [69].

Tretinoin nanosuspension and nanoemulsion was investigated to check the feasibility for dermal and transdermal targeting. In vitro diffusion studies and in vivo studies in pigs showed that tretinoin transdermal permeation was superior in nano-emulsion formulation and photostability was considerably improved in nanosuspension [43].

Transdermal delivery of methotrexate coated with non-ionic surfactant and stabilized by l-arginine was tested by solid-in-oil technique. The transdermal efficiency for the solid-in-oil nanosuspension was 2–3 folds than the control aqueous solution. On account of smaller particle size (< 100 nm), the oil-based nanosuspension is more efficient in permeating the stratum corneum [91]. The performance of ibuprofen nanosuspension and effect of varying concentrations of the solubilizer, Vitamin E TPGS to increase the permeation rate across the skin has been investigated. It was found that the entire transdermal permeation across the skin was mainly dictated by the size of nanoparticles and concentration of solubilizer [20].

#### Future perspectives and challenges

The future prospects of nanosuspension are encouraging since they can contribute as a valuable tool for product development scientists to overcome various formulation and drug delivery challenges particularly with intractable drugs. Regardless of the several published research in the area of nanosuspension, the critical aspects of stability issue pertaining to nanosuspension is still unresolved. The stabilization capability of the electrostatic and steric stabilizers and its relationship with the properties APIs, attainable maximum particle size and resulting physical stability are the critical factors need to be further investigated. Advancement in biotechnology and aid of modification tools such as antibody-drug conjugate and nanobodies will probably create subcutaneously administered, high concentration monoclonal (mAb) and biosimilar products with enhanced biopharmaceutical and safety characteristics. Recently, Johnston et al. [33] have developed biologically active, nanocluster dispersions of antibodies in solution (~ 250 mg/ml) using carbohydrate stabilizers like trehalose. Future development of enabling technologies like nanosuspension will provide technical solutions to many formulation challenges currently faced by protein and peptide based drugs.

## Conclusions

Nanosuspension can be considered as the best formulation option for inflexible hydrophobic drugs restricted by high log P, molecular weight, melting point and dose. Conventional size reduction operations such as wet milling and homogenization and formulation approaches such as precipitation, emulsion-solvent evaporation, solvent diffusion and microemulsion techniques can be successfully utilized to prepare and scale-up nanosuspensions. Substantial improvement of bioavailability due to increased saturation and intrinsic solubility, appreciable mucoadhesivity, adaptability for surface modification in drug targeting have broadly expanded the scope of this novel formulation. Nanosuspension has the potential to consider as a valuable tool for formulation scientist to overcome many formulation and drug delivery challenges pertaining to various drug entities. The application of nanosuspensions in oral, ocular and pulmonary drug delivery systems have been extensively researched during the last few decades. Further, utilization of nanosuspensions in other drug delivery systems such as brain, topical, buccal, nasal and transdermal routes are under extensive investigation. Though nanosuspension has received serious consideration from pharmaceutical scientists, the exact mechanisms of stabilization, solidifications and redispersibility of dried nanosuspension are yet to be explored.

## Data Availability

Please contact author for data requests.

## Abbreviations

AUC: Area under plasma-drug concentration time profile
BCS: Biopharmaceutical Classification System
Cmax: Maximum concentration of drug
EPR: Enhanced Permeation and Retention Effects
FDA: Food and Drug Administration
GRAS: Generally regarded as safe
HEC: Hydroxyethyl cellulose
HPC: Hydroxypropyl cellulose
HPMC: Hydroxypropyl methyl cellulose
PVA: Polyvinyl alcohol
Vitamin E TPGS: D-α-Tocopheryl polyethylene glycol 1000 succinate
XRD: X-Ray diffractometer
